# Supplementary material for: Patient-Reported Functional Outcomes and Quality of Life After Contact X-Ray Brachytherapy (CXB) in Organ-Preserving Management of Rectal Cancer
Source: Cancers (Basel). 2025 May 3;17(9):1560. doi: 10.3390/cancers17091560 (PMC12071122; doi:10.3390/cancers17091560)
Supplement: Supplementary file 1 [file cancers-17-01560-s001.zip › Supplementary table S1.pdf]

Supplementary table s1: Detailed oncological outcomes of patients who received Contact X-ray Brachytherapy with various intent

Outcome	CXB intent												
	De novo (n=12)			Residual (n=26)						Regrowth (n=15)			
	T1-2N0M0 (n=8)	T3N0M0 (n=1)	T1-2N1M0 (n=3)	T1-2N0M0 (n=5)	T3N0M0 (n=6)	T4N0M0 (n=2)	T1-2N1M0 (n=2)	T3N1-2M0 (n=10)	TxNxM0 (n=1)	T1-2N0M0 (n=4)	T3N0M0 (n=6)	T3N1-2M0 (n=3)	TxNxM0 (n=2)
**Complete response**	6	1	3	5	3	1	0	6	1	1	1	1	2
**Residual disease**	2	0	0	0	3	1	1	4	0	3	4	2	0
**Local regrowth**	0	0	0	0	0	0	1	0	0	0	1	0	0
**Distant relapse**	0	0	0	1	2	0	0	1	0	1	0	1	0
**Pelvic nodal relapse**	1	0	0	0	0	0	0	0	0	0	0	0	0
