# Supplementary material for: Patient-Reported Functional Outcomes and Quality of Life After Contact X-Ray Brachytherapy (CXB) in Organ-Preserving Management of Rectal Cancer
Source: Cancers (Basel). 2025 May 3;17(9):1560. doi: 10.3390/cancers17091560 (PMC12071122; doi:10.3390/cancers17091560)
Supplement: Supplementary file 1 [file cancers-17-01560-s001.zip › Supplementary table S2.pdf]

*Supplementary table s2: Longitudinal analyses using Linear Mixed Effect (LME) models for questionnaire scores. The coefficient shown is the association between time and symptom domain. The multivariate model adjusts for treatment intent, EBRT regimen, and disease status.*

| EORTC-QLQ-CR29 | | | | |
|---|---|---|---|---|
| **Bowel symptoms** | **Adjustment variables** | **Coefficient** | **P value** | **95% CI** |
| Abdominal pain | Unadjusted | -1.85 | **0.03** | -3.49, -0.20 |
| | treatment intent | -1.87 | **0.03** | -3.51, -0.22 |
| | EBRT regimen | -1.85 | **0.03** | -3.49, -0.20 |
| | disease status | -1.87 | **0.05** | -3.73, -0.03 |
| | Multivariate | -1.85 | 0.052 | -3.72, -0.01 |
| Buttock pain | Unadjusted | 0.78 | 0.52 | -1.61, 3.17 |
| | treatment intent | 0.68 | 0.57 | -1.70, 3.07 |
| | EBRT regimen | 0.66 | 0.59 | -1.72, 3.05 |
| | disease status | -0.12 | 0.92 | -2.82, 2.57 |
| | Multivariate | -0.21 | 0.88 | -2.92, 2.45 |
| Bloated feeling | Unadjusted | -2.36 | 0.09 | -5.07, 0.35 |
| | treatment intent | -2.43 | 0.08 | -5.14, 0.29 |
| | EBRT regimen | -2.48 | 0.07 | -5.19, 0.22 |
| | disease status | -2.51 | 0.11 | -5.60, 0.55 |
| | Multivariate | -2.62 | 0.10 | -5.72, 0.43 |
| Blood and mucous stool | Unadjusted | -0.18 | 0.88 | -2.40, 2.06 |
| | treatment intent | -0.24 | 0.82 | -2.47, 1.99 |
| | EBRT regimen | -0.28 | 0.81 | -2.50, 1.96 |
| | disease status | -1.88 | 0.14 | -4.41, 0.62 |
| | Multivariate | -1.99 | 0.12 | -4.53, 0.49 |
| Flatulence | Unadjusted | -5.93 | **<0.001** | -9.16, -2.71 |
| | treatment intent | -6.06 | **<0.001** | -9.30, -2.84 |
| | EBRT regimen | -6.05 | **<0.001** | -9.29, -2.84 |
| | disease status | -7.63 | **<0.001** | -11.26, -4.02 |
| | Multivariate | -7.70 | **<0.001** | -11.35, -4.11 |
| Faecal incontinence | Unadjusted | -0.23 | 0.84 | -2.64, 2.17 |
| | treatment intent | -0.36 | 0.77 | -2.75, 2.04 |
| | EBRT regimen | -0.32 | 0.80 | -2.72, 2.10 |
| | disease status | -1.61 | 0.24 | -4.34, 1.11 |
| | Multivariate | -1.69 | 0.22 | -4.42, 0.10 |
| Sore skin | Unadjusted | -0.53 | 0.55 | -2.27, 1.22 |
| | treatment intent | -0.57 | 0.51 | -2.31, 1.17 |
| | EBRT regimen | 2.19 | 0.52 | -4.43, 8.84 |
| | disease status | -1.34 | 0.18 | -3.30, 0.63 |
| | Multivariate | -1.33 | 0.19 | -3.30, 0.63 |
| Stool frequency | Unadjusted | -1.06 | 0.17 | -2.55, 0.45 |
| | treatment intent | -1.11 | 0.15 | -2.61, 0.39 |
| | EBRT regimen | -1.15 | 0.13 | -2.65, 0.35 |
| | disease status | -1.44 | 0.09 | -3.16, 0.26 |
| | Multivariate | -1.57 | 0.07 | -3.29, 0.12 |
| Embarrassment | Unadjusted | -0.09 | 0.95 | -2.96, 2.79 |
| | treatment intent | -0.17 | 0.90 | -3.04, 2.70 |
| | EBRT regimen | -0.20 | 0.89 | -3.07, 2.67 |

| | | | | |
|---|---|---|---|---|
| | disease status | -1.99 | 0.23 | -5.29, 1.26 |
| | Multivariate | -2.16 | 0.19 | -5.48, 1.05 |

| **Urinary symptoms** | | **Coefficient** | **P value** | **95% CI** |
|---|---|---|---|---|
| Urinary frequency | Unadjusted | -4.63 | **<0.009** | -6.90, -2.37 |
| | treatment intent | -4.63 | **<0.009** | -6.90, -2.37 |
| | EBRT regimen | -4.65 | **<0.009** | -6.93, -2.39 |
| | disease status | -5.82 | **<0.001** | -8.36, -3.27 |
| | Multivariate | -5.84 | **<0.001** | -8.40, -3.30 |
| Urinary incontinence | Unadjusted | -0.07 | 0.95 | -2.53, 2.47 |
| | treatment intent | -0.36 | 0.80 | -2.70, 2.94 |
| | EBRT regimen | -0.22 | 0.82 | -2.75, 2.94 |
| | disease status | -0.06 | 0.89 | -2.82, 2.67 |
| | Multivariate | -0.13 | 0.71 | -1.97, 2.47 |
| Dysuria | Unadjusted | -0.17 | 0.53 | -0.69, 0.36 |
| | treatment intent | -0.18 | 0.49 | -0.71, 0.34 |
| | EBRT regimen | -0.18 | 0.52 | -0.70, 0.35 |
| | disease status | -0.13 | 0.67 | -0.72, 0.46 |
| | Multivariate | -0.11 | 0.71 | -2.85, 1.56 |
| **Sexual symptoms** | | **Coefficient** | **P value** | **95% CI** |
| Impotence/ Dyspareunia | Unadjusted | 0.28 | 0.80 | -1.94, 2.50 |
| | treatment intent | 0.26 | 0.82 | -1.97, 2.47 |
| | EBRT regimen | 0.28 | 0.80 | -1.94, 2.50 |
| | disease status | -0.07 | 0.95 | -2.57, 2.40 |
| | Multivariate | -0.06 | 0.96 | -2.56, 2.42 |
| **Functional scales** | | **Coefficient** | **P value** | **95% CI** |
| Anxiety | Unadjusted | -2.51 | 0.055 | -5.07, 0.04 |
| | treatment intent | -2.47 | 0.06 | -5.03, 0.08 |
| | EBRT regimen | -2.37 | 0.07 | -4.91, 0.18 |
| | disease status | -0.36 | 0.80 | -3.19, 2.49 |
| | Multivariate | -0.18 | 0.90 | -2.99, 2.68 |
| Weight | Unadjusted | 3.35 | **0.01** | 0.82, 5.86 |
| | treatment intent | 3.34 | **0.01** | 0.81, 5.85 |
| | EBRT regimen | 3.47 | **0.007** | 0.96, 5.99 |
| | disease status | 5.37 | **<0.001** | 2.61, 8.10 |
| | Multivariate | 5.52 | **<0.001** | 2.75, 8.22 |
| Body image | Unadjusted | 0.83 | 0.32 | -0.81, 2.46 |
| | treatment intent | 0.84 | 0.32 | -0.80, 2.48 |
| | EBRT regimen | 0.89 | 0.28 | -0.76, 2.52 |
| | disease status | 1.93 | **0.03** | 0.15, 3.71 |
| | Multivariate | 2.00 | **0.03** | 0.21, 3.76 |
| Sexual interest | Unadjusted | -0.18 | 0.89 | -2.74, 2.35 |
| | treatment intent | -0.12 | 0.92 | -2.68, 2.41 |
| | EBRT regimen | -0.22 | 0.86 | -2.78, 2.30 |
| | disease status | 0.97 | 0.50 | -1.85, 3.78 |
| | Multivariate | 0.96 | 0.50 | -1.87, 3.75 |
| **HADS** | | | | |

| | | Coefficient | P value | 95% CI |
|---|---|---|---|---|
| Anxiety | Unadjusted | -0.29 | 0.125 | -0.65, 0.08 |
| | treatment intent | -0.29 | 0.12 | -0.65, 0.07 |
| | EBRT regimen | -0.28 | 0.12 | -0.65, 0.08 |
| | disease status | -0.39 | 0.09 | -0.09, 3.29 |
| | Multivariate | -0.41 | 0.07 | -0.85, 0.04 |
| Depression | Unadjusted | 0.04 | 0.80 | -0.26, 0.34 |
| | treatment intent | 0.04 | 0.80 | -0.26, 0.34 |
| | EBRT regimen | 0.04 | 0.80 | -0.26,0.34 |
| | disease status | -0.01 | 0.98 | -0.37, 0.36 |
| | Multivariate | -0.02 | 0.92 | -0.39, 0.35 |
| | | **EQ-5D 3L** | | |
| | | **Coefficient** | **P value** | **95% CI** |
| Index score | Unadjusted | 0.07 | 0.24 | -0.04, 0.17 |
| | treatment intent | 0.06 | 0.25 | -0.04, 0.17 |
| | EBRT regimen | -0.01 | 0.84 | -0.13, 0.11 |
| | disease status | -0.01 | 0.84 | -0.13, 0.11 |
| | Multivariate | -0.01 | 0.84 | -0.13, 0.11 |
| EQ-VAS | Unadjusted | 1.84 | **0.01** | 0.43, 3.26 |
| | treatment intent | 1.84 | **0.01** | 0.43, 3.27 |
| | EBRT regimen | 1.86 | **0.01** | 0.45, 3.28 |
| | disease status | 2.91 | **0.0004** | 1.34, 4.49 |
| | Multivariate | 2.92 | **0.0004** | 1.36, 4.51 |
