# Supplementary figures and images for: Patient-Reported Functional Outcomes and Quality of Life After Contact X-Ray Brachytherapy (CXB) in Organ-Preserving Management of Rectal Cancer
Source: Cancers (Basel). 2025 May 3;17(9):1560. doi: 10.3390/cancers17091560 (PMC12071122; doi:10.3390/cancers17091560)

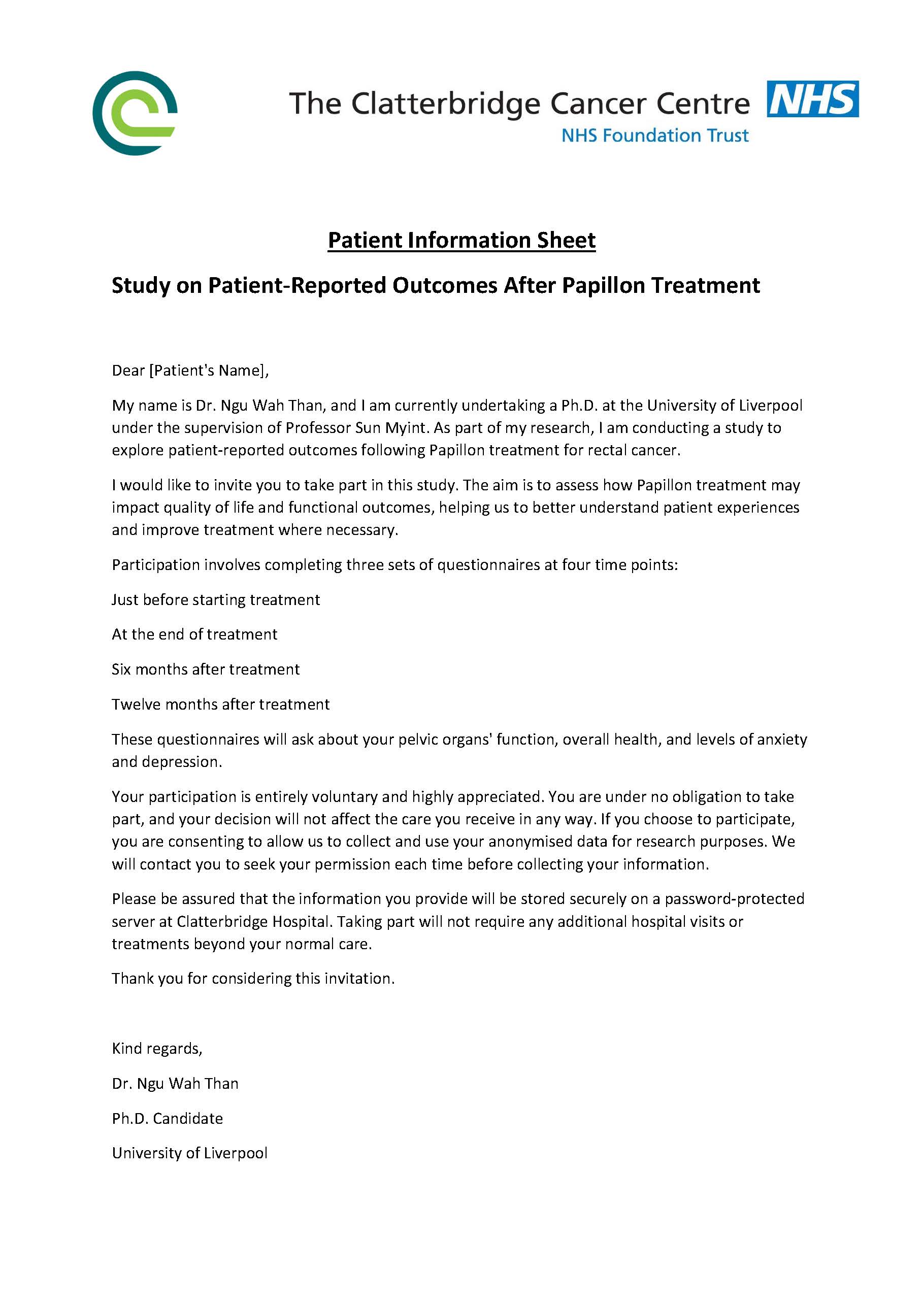

Supplement: Supplementary file 1 [file cancers-17-01560-s001.zip › Supplementary figure S1.jpg]
